# New Appliances and Things Medical

**Published:** 1904-01-23

**Authors:** 


### NEW APPLIANCES AND THINGS MEDICAL.

[We shall be glad to receive, at our Office, 28 & 29 Southampton Street Strand, London, W.C., from the manufacturers, specimens of all new preparations and appliances which may be brought out from time to time.]

### STERILISED CREAM.

(FUSSELL AND COMPANY, 4 MONUMENT STREET, LONDON, E.C.)

WE have examined a sample of this cream and find it to be pure and of good flavour. No preservatives have been added, yet, owing to its complete sterilisation, it will keep untainted for a lengthened period of time. To some invalids and convalescents, especially those of tender years, cream is an extremely useful article of diet, and it will be a boon to many practitioners to know where they can procure for their patients a cream which has all the advantages of the fresh product without the chance of bacterial contamination or the presence of harmful antiseptics. For this purpose we have much pleasure in recommending Fussell's Sterilised Cream. It can be obtained in sixpenny, ninepenny, or shilling tins, and in half-gallon or gallon sizes also.

